# Salinomycin Induces Autophagy in Colon and Breast Cancer Cells with Concomitant Generation of Reactive Oxygen Species

**DOI:** 10.1371/journal.pone.0044132

**Published:** 2012-09-19

**Authors:** Berlinda Verdoodt, Markus Vogt, Inge Schmitz, Sven-Thorsten Liffers, Andrea Tannapfel, Alireza Mirmohammadsadegh

**Affiliations:** Institute of Pathology, Ruhr-University Bochum, Bochum, Germany; Juntendo University School of Medicine, Japan

## Abstract

**Background:**

Salinomycin is a polyether ionophore antibiotic that has recently been shown to induce cell death in human cancer cells displaying multiple mechanisms of drug resistance. The underlying mechanisms leading to cell death after salinomycin treatment have not been well characterized. We therefore investigated the role of salinomycin in caspase dependent and independent cell death in colon cancer (SW480, SW620, RKO) and breast cancer cell lines (MCF-7, T47D, MDA-MB-453).

**Methodology/Principal Findings:**

We detected features of apoptosis in all cell lines tested, but the executor caspases 3 and 7 were only strongly activated in RKO and MDA-MB-453 cells. MCF-7 and SW620 cells instead presented features of autophagy such as cytoplasmic vacuolization and LC3 processing. Caspase proficient cell lines activated autophagy at lower salinomycin concentrations and before the onset of caspase activation. Salinomycin also led to the formation of reactive oxygen species (ROS) eliciting JNK activation and induction of the transcription factor *JUN*. Salinomycin mediated cell death could be partially inhibited by the free radical scavenger N-acetyl-cysteine, implicating ROS formation in the mechanism of salinomycin toxicity.

**Conclusions:**

Our data indicate that, in addition to its previously reported induction of caspase dependent apoptosis, the initiation of autophagy is an important and early effect of salinomycin in tumor cells.

## Introduction

Salinomycin, a polyether antibiotic which acts as a highly selective potassium ionophore, and which is widely used as an anticoccidial drug, is now being increasingly recognized as a novel and effective anti-cancer agent [1]. This chemotherapeutic agent induces massive apoptosis in human cancer cells of different origin, but not in normal cells such as human T lymphocytes [2]. Furthermore, salinomycin was able to reduce the proportion of epithelial cancer stem cells by more than 100 fold relative to paclitaxel. It was also effective against tumor cells exhibiting ABC transporter-mediated multidrug resistance, and had an inhibitory effect on the multidrug efflux transporter P-glycoprotein (P-gp) [3], [4]. However, the molecular mechanism underlying salinomycin induced cell death still remains unclear. In the human acute lymphoblastic leukemia cell line Molt-4 the general caspase inhibitor N-benzyloxycarbonyl-L-valyl-L-alanyl-L-aspartate fluoromethylketone (Z-VADfmk) did not diminish cell death by salinomycin, indicating that caspase-independent mechanisms may also play a role [2]. Another possible pathway leading to cell death is autophagy, characterized by the appearance of vacuoles in the cytoplasm and induction of the BH3 protein Beclin-1, as well as autophagy genes (ATG) [5]. Although this pathway can be induced to protect cells from death during nutrient starvation, it also acts as an alternative cell death pathway, for instance after treatment of cells with a compound that causes loss of mitochondrial membrane potential [6], in BAK/BAX knock-out mouse embryonic fibroblasts [7], or after chemical inhibition of caspases [8].

Autophagy induction after mitochondrial damage has recently been found to be accompanied by the production of reactive oxygen species (ROS) [5], [6]. ROS regulate autophagy by multiple mechanisms that involve, amongst others, increased expression of the ATG4 protein [9], inactivation of catalase [10] and the mitochondrial electron transport chain [5]. Downstream targets of ROS in the induction of cell death are the mitogen activated protein kinase (*MAPK*) pathways, via induction of apoptosis signal regulated kinase 1 (*ASK1*) [11], and inactivation of MAPK phosphatases [12]. This subsequently leads to the phosphorylation of JNK, and of its target the transcription factor JUN. JNK activation may promote autophagy through induction of *ATG7* [6], or by phosphorylation of BCL2, which leads to dissociation of BCL2 from Beclin-1 [13]. Moreover, inhibition of *JUN* by siRNA was found to inhibit autophagy after Z-VAD-fmk treatment of L929 fibrosarcoma cells [10].

Therefore, in the present study we investigated the relative importance of caspase–dependent and independent cell death pathways after salinomycin treatment of colon and breast cancer cell lines. We observed an induction of autophagy after salinomycin treatment, with concomitant formation of the ROS, namely O_2_• and H_2_O_2_ and activation of the JNK pathway. Inhibition of ROS with the free radical scavenger N-acetyl cysteine (NAC) decreased the toxicity of salinomycin. To our current knowledge, this is the first report of autophagy induction by salinomycin.

## Results

### Effect of salinomycin on cell viability and colony formation in colon and breast cancer cell lines

**Figure 1:**
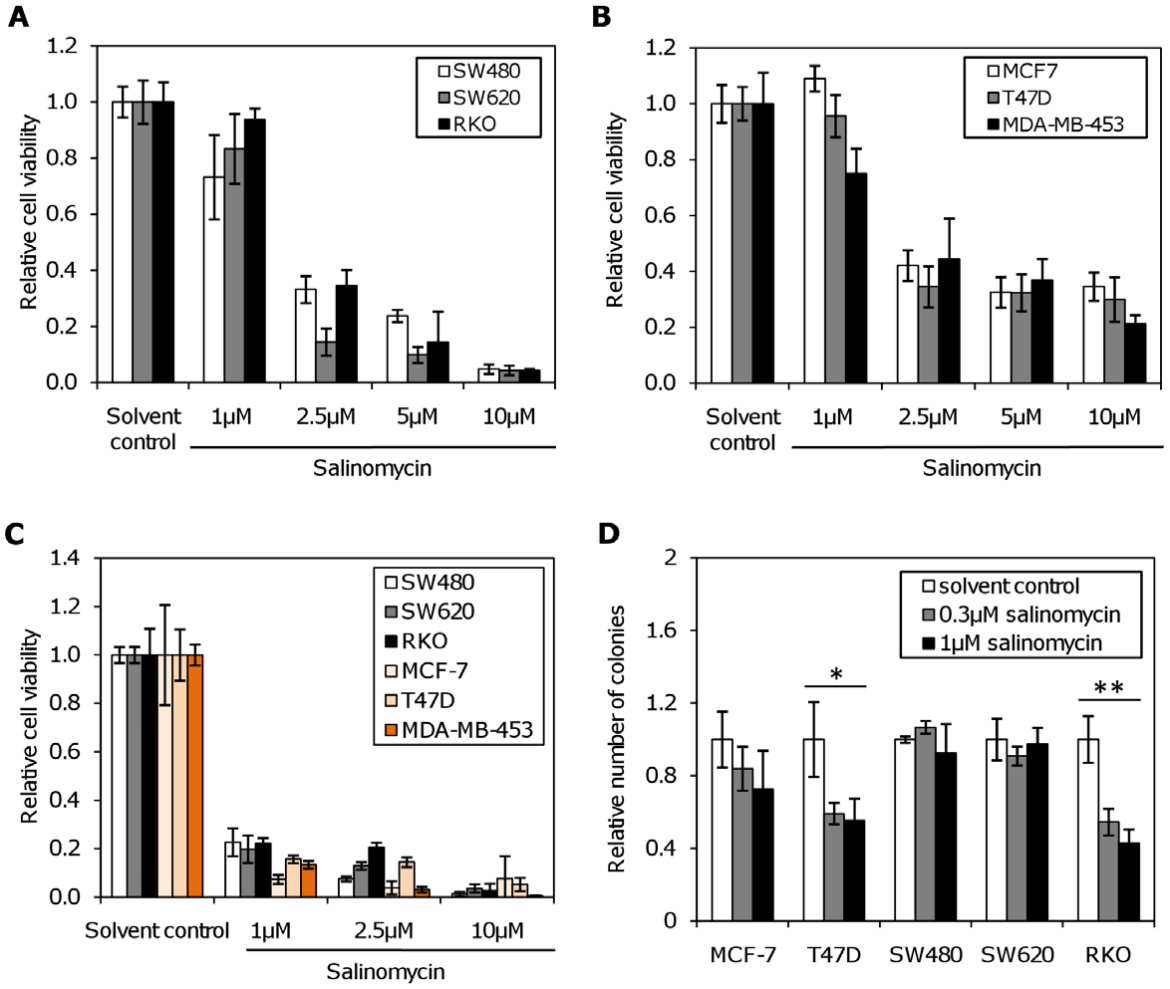
Effect of salinomycin on cell viability in colon and breast cancer cell lines. The cell viability was determined by MTT and colony forming assays. (**A**) Colon cancer cell lines, 72 hours post treatment (p<0.001, unifactorial ANOVA); (**B**) breast cancer cell lines, 72 hours post treatment (p<0.001, unifactorial ANOVA). (**C**) Cell viability assay performed 6 days post treatment. Colon and breast cancer cell lines were treated with the indicated concentrations of salinomycin. Salinomycin reduced the cell viability in a time and concentration dependent manner (p<0.001, unifactorial ANOVA). (**D**) Sensitivity to salinomycin in the colony forming assay. Assays were performed in triplicates. After incubation for 10 days, the plates were stained with crystal violet. The reduction of colony number depended on the salinomycin concentration and the cell line (*: p<0.05; **: p<0.01 by unifactorial anova).

We first examined the impact of salinomycin on cell viability and colony formation in a panel of colon (RKO, SW480, SW620), and breast cancer cell lines (MCF-7, MDA-MB-453, T47D). Rapid chemosensitivity testing of human colon and breast cancer cell lines using the MTT assay showed a significant dose-dependent decrease in cell viability. Interestingly, the colorectal carcinoma cell lines demonstrated greater sensitivity to salinomycin than the breast cancer cell lines. At the highest concentration (10 µM), the cell viability decreased by 95% in comparison to the solvent control in colorectal cancer (CRC) cell lines (Figure 1A), but only by maximally 80% in breast cancer cell lines (Figure 1B). The decrease in viability was highly significant (p<0.001 by unifactorial ANOVA for all cell lines), Post-hoc testing reveals that the difference between solvent control and salinomycin treatment is significant for all concentrations >1 µM for all tested cell lines after 72 hrs; the difference is significant for 1 µM only in MDA-MB-453. The results of the MTT assay correlated well with the loss of membrane integrity after salinomycin treatment as measured by the ViaCount assay (Figure S1A, S1B). This further supports that the decrease in MTT metabolisation is not due to decreased metabolic activity of still viable cells. Six days post treatment, cell viability diminished by more than 80% even at lower concentrations (2.5 µM salinomycin) in both breast and colon cancer cells, and the cells were not able to recover (p<0.001 by unifactorial ANOVA for all cell lines, Figure 1C). Supporting these results, in a colony forming assay RKO cells were the most sensitive to lower salinomycin concentrations, with a ∼40% (p<0.001, unifactorial ANOVA) reduction of colony formation at 0.3 µM and at 1 µM of salinomycin. In this assay, T47D was the most sensitive cell line (p<0.02) among the tested breast cancer cell lines (Figure 1D). It was not possible to carry out this assay with the cell line MDA-MB-453, as these cells have a colony-forming efficiency of 0% at any meaningful plating density (of up to 1500 cells per well in a six-well plate).

### Salinomycin induces apoptosis in breast and colon cancer cell lines

**Figure 2:**
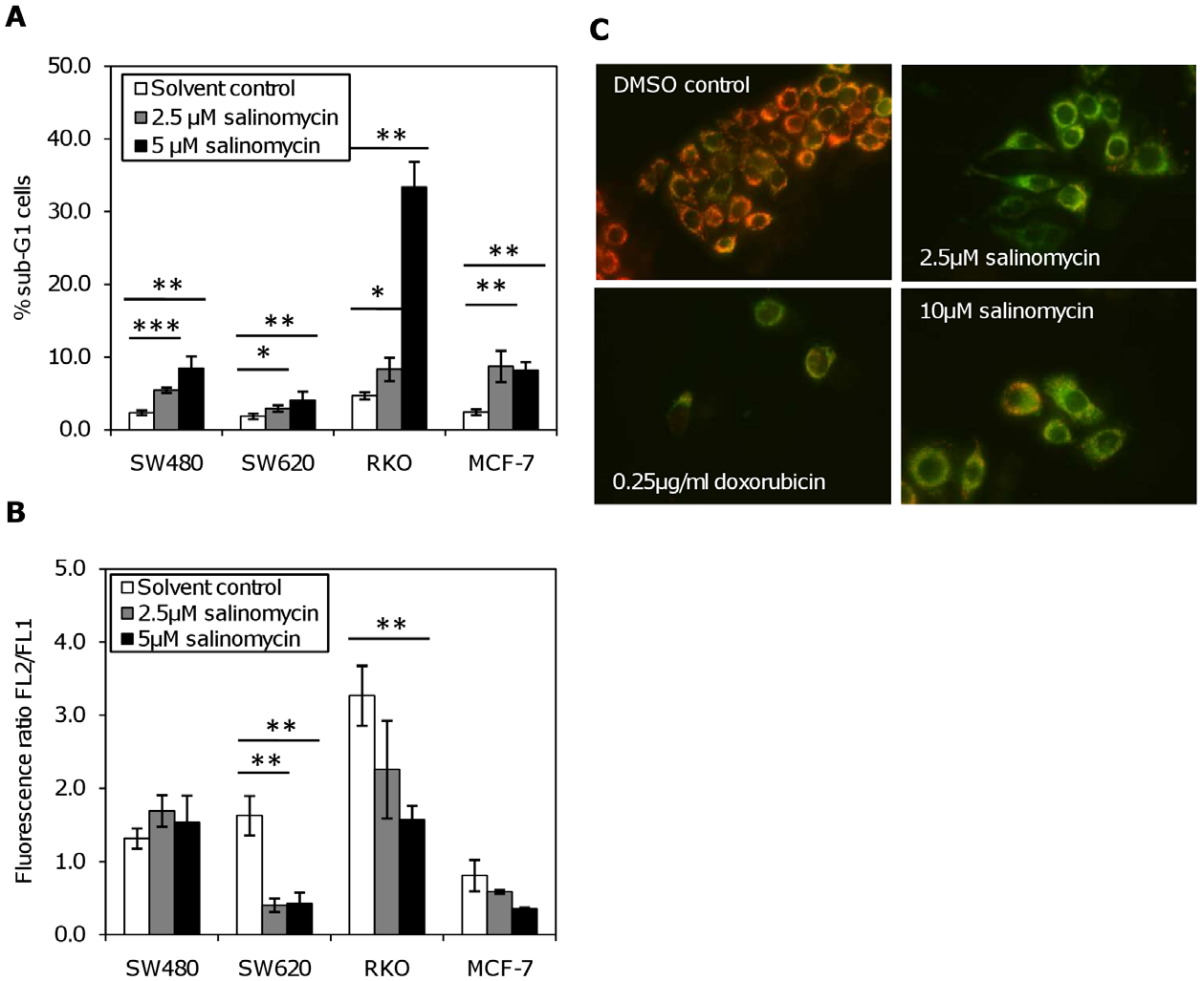
Induction of cell death by salinomycin in colon and breast cancer cell lines. (**A**) DNA fragmentation is induced after 48 hours of salinomycin treatment. DNA fragmentation was measured by flow cytometry as a sub-G1 cell fraction after propidium iodide staining (*: p<0.05; **: p<0.01; ***: p<0.001 by t-test). (**B**) Measurement of the mitochondrial potential using JC-1 staining, determined as the ratio of red (FL2, JC1-aggregates) and green fluorescence (FL1, monomeric form) (*: p<0.05; **: p<0.01; ***: p<0.001 by t-test). (**C**) Staining of colon cancer cell line SW480 with JC-1 visualization *via* fluorescence microscopy. Cells without salinomycin treatment show orange fluorescence, indicating an intact mitochondrial potential. Apoptotic cells with decreased mitochondrial potential show green fluorescence.

To investigate the mechanism of cell death by salinomycin we used different methods for the detection of apoptosis. First we evaluated DNA fragmentation by flow cytometry. Forty-eight hours post treatment, we observed a concentration dependent increase in the fraction of sub-G1 cells in all tested cell lines (Figure 2A). The RKO cells showed a seven-fold increase in the sub-G1 fraction in comparison to the respective solvent control, making it the most sensitive cell line in this assay. In contrast to their sensitivity in the MTT and colony formation assays, SW620 cells presented the lowest fraction of sub-G1-phase cells.

Next we examined whether salinomycin-induced cell death was mediated through the mitochondrial pathway, by determining its effect on the mitochondrial membrane potential (Δψm) using the cationic dye JC-1. The loss of mitochondrial membrane potential leads to a shift in the fluorescence spectrum of JC-1 from an aggregated form with orange fluorescence to a monomeric form with green fluorescence. RKO, MCF-7, SW480, and SW620 cells were treated with salinomycin for 48 hours, leading to a pronounced Δψm, which was significant in SW620 and RKO cells. In this assay, SW620 cells were most sensitive to salinomycin, in contrast to the results obtained with the DNA fragmentation (Figure 2B, 2C).

### Is salinomycin mediated cell death caspase dependent?

**Figure 3:**
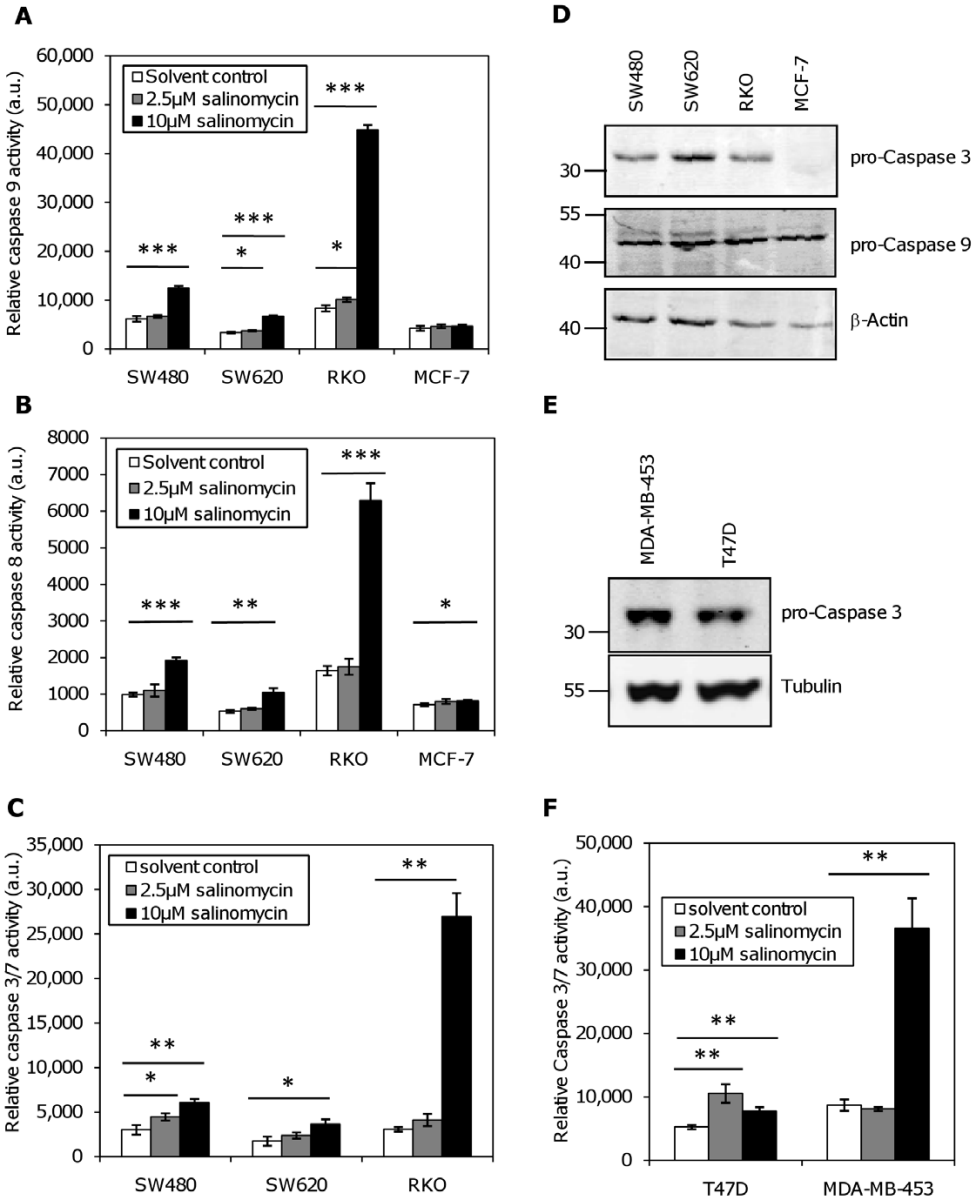
Impact of salinomycin on caspase activation. Caspase activities were measured after treatment of breast and colon cancer cell lines with salinomycin for 28 hours. (**A**) Caspase 9 activity measured with Z-LEHD-aminoluciferin as a substrate; (**B**) Caspase 8 activity measured with Z-LETD-aminoluciferin as a substrate; (**C**) Caspase 3/7 activity measured in colon cancer cell lines with Z-DEVD-aminoluciferin as a substrate. Measurements were in MCF-7 cells were omitted as these cells are caspase 3-negative. Significant increases in measured luminescence in comparison to the untreated cells are indicated in the graphs (*: p<0.05; **: p<0.01, ***: p<0.001 by t-test). RKO cells were most sensitive to salinomycin treatment for all the measured caspases. (**D**) Western blot analysis of caspase status. Procaspase 3 and procaspase 9 expression were determined in SW480, SW620, RKO, and MCF-7 cells. β-Actin was used as a loading control. (**E**) Caspase 3 status in T47D and MDA-MB-453 cells. Procaspase 3 expression was determined by Western blot; α-Tubulin was used as a loading control. (**F**) Caspase 3/7 activity measured in breast cancer cell lines with Z-DEVD-aminoluciferin as a substrate. Caspase 3 activity was not measured in MCF-7 as these cells are caspase 3-negative.

As the loss of Δψm and DNA fragmentation were indicative of apoptosis, we measured the activities of the initiator caspases 8 and 9, as well as the effector caspases 3 and 7. For this, an assay based on the cleavage of a luciferin-linked caspase substrate was used. The resulting light emission is proportional to the caspase activity in the sample. In the presence of salinomycin caspase 9 activity was only clearly induced in RKO and SW480 cells; only minimal caspase 9 activation was seen in MCF-7 and SW620, despite expression of the protein (Figure 3A and 3D), and cytochrome C release from the mitochondria after salinomycin treatment in MCF-7 (Figure S1C, S1D).

Caspases 8 (Figure 3B) and caspase 3/7 (Figure 3C) activation follow the same pattern as caspase 9, i.e. only RKO and SW480 exhibited significant caspase activity; SW620 did not induce caspase 3/7 activity after salinomycin treatment, although it does express both caspase 9 and 3 (Figure 3C, 3D). As expected, no caspase 3/7 activity could be detected in caspase 3-negative MCF-7 cells (Figure 3D). On the other hand, both caspase-3 expressing breast cancer cell lines, T47D and MDA-MB-453, show a significant induction of caspase 3 activity. Especially MDA-MB-453 is very sensitive to caspase activation by high doses of salinomycin (Figure 3E, 3F). Caspase activities were measured 28 hours after salinomycin treatment, because even in the most salinomycin sensitive cell line RKO, caspase activation starts relatively late after exposure. A time course experiment in this cell line showed a continuous increase of caspase activity from 8 to 24 hours after treatment (Figure S2A). Nevertheless, even at the earliest time point in this series, viability had decreased to only 30% of the level of the control cells (Figure S2B). The relatively weak activation of caspases in contrast to the toxicity in the MTT and colony forming assays indicate that a caspase independent cell death pathway must also be activated by salinomycin treatment.

### Cell death by salinomycin proceeds via the autophagic pathway

**Figure 4:**
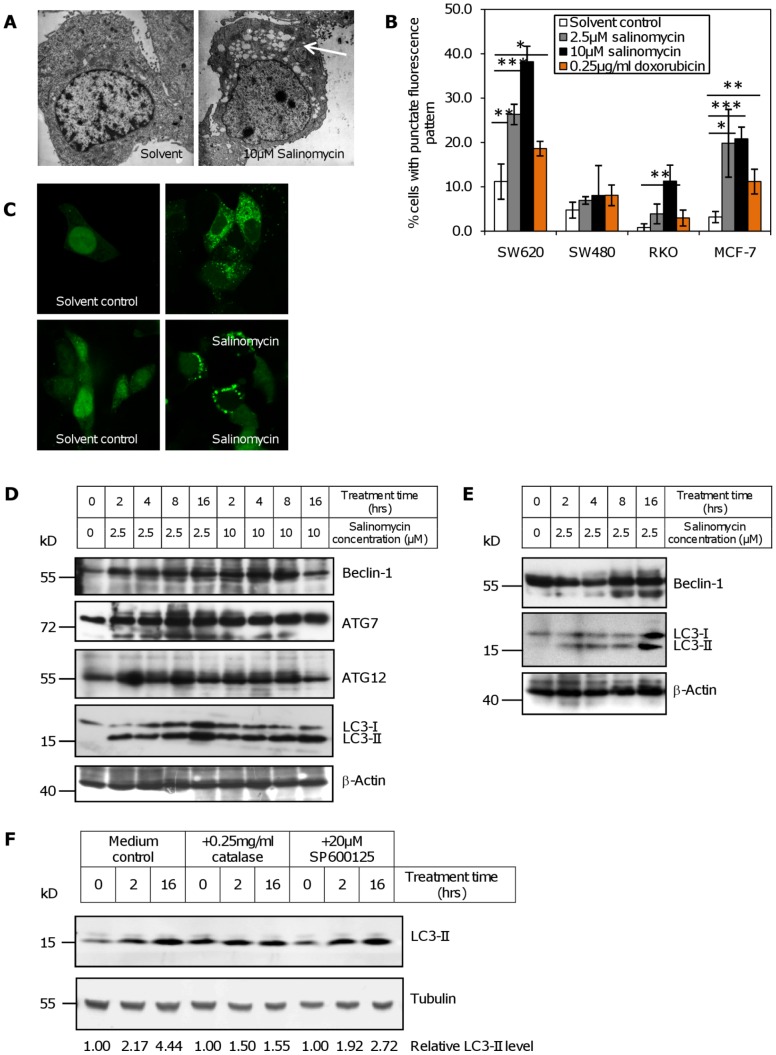
Induction of autophagy by salinomycin. (**A**) Electron microscopic images of MCF-7 cells 48 hours after treatment with 10 µM salinomycin or solvent control. Note the vacuoles formed in the cytoplasm (arrow). (**B**) Salinomycin induced uptake of monodansylcadaverine (MDC) in SW620, RKO and MCF-7 cells 8 hours after salinomycin treatment, in comparison to doxorubicin. Cells with a punctate staining pattern were counted. (*: p<0.05; **: p<0.01, ***: p<0.001 by t-test, compared to the solvent control). (**C**) Confocal microscopy of cells transfected with GFP-LC3 16 hours after treatment as indicated (sali: 2.5 µM salinomycin). Upper row: MCF-7, bottom row: SW620. (**D**) Induction of the autophagy-related genes *Beclin-1*, *ATG7*, and *ATG12*, and processing of LC3B after salinomycin treatment of MCF-7. Cells were treated for the indicated durations with 2.5 µM or 10 µM salinomycin, and analyzed for autophagy-specific markers by Western blot. β-actin was used as a loading control. (**E**) Expression of the autophagy-related gene *Beclin-1*, and processing of LC3B after salinomycin treatment in SW620. Cells were treated for the indicated durations with 2.5 µM salinomycin, and analyzed for autophagy-specific markers by Western blot. β-Actin was used as a loading control. (**F**) Processing of LC3 in SW480 after salinomycin treatment in the presence or absence of 0.25 mg/ml catalase and 20 µM SP600125. Cells were treated with 2.5 µM salinomycin for the indicated duration; the relative level of LC3-II in comparison to tubulin is indicated below the figure.

A distinct phenotype of MCF-7 cells treated with salinomycin is the formation of multiple vacuoles, indicative for the induction of autophagy. By electron microscopy we observed numerous autophagic vacuoles 48 hours post exposure of MCF-7 cells to 10 µM salinomycin (Figure 4A). For further evidence for salinomycin mediated autophagy, we tested this cell line, and the CRC cell lines RKO, SW480, and SW620 for increased uptake of monodansyl cadaverine (MDC), a specific marker for autophagy [14]. Especially MCF-7 and SW620 show a significant increase in MDC uptake (Figure 4B) eight hours after salinomycin treatment, evident as an increased fraction of cells with punctate staining. This was not so clearly apparent after doxorubicin treatment. In RKO and SW480 cells, which strongly induce caspase activity, a smaller number of cells show a clearly punctate pattern of fluorescence, but a similar trend was observed. These results were confirmed in MCF-7 and SW620 cells transiently transfected with pEGFP-LC3B [15]. Both cell lines showed an increased vacuolar localization of the GFP signal after treatment with 2.5 µM salinomycin for 16 hours (Figure 4C, right panels), in comparison to the solvent control (Figure 4C, left panels). Concordant with the results from electron microscopy, MDC incorporation, and GFP-LC3 staining, an increased expression of Beclin-1, ATG7 and ATG12 was detected by western blot 0.5–16 hours after salinomycin treatment of MCF-7 cells. Processing of LC3 to the lower apparent molecular weight autophagy specific form LC3-II was observed as early as 2 hours after treatment of both MCF-7 (Figure 4D) and SW620 cells (Figure 4E). Together with a small increase in MDC uptake, a more pronounced increase in LC3 processing also occurred in SW480 after salinomycin treatment (Figure 4F).

In contrast to the result obtained from MCF-7 cells, no increase in Beclin-1 levels could be seen in SW620 (Figure 4E). Consistent with the increases in protein expression, a strong induction of *Beclin-1* mRNA was also observed 4 hours after salinomycin treatment in MCF-7 cells; in addition dose-dependent inductions of *ATG5*, *ATG7*, and *ATG12* occurred at the same time point (Figure S3A, S3B).

**Figure 5:**
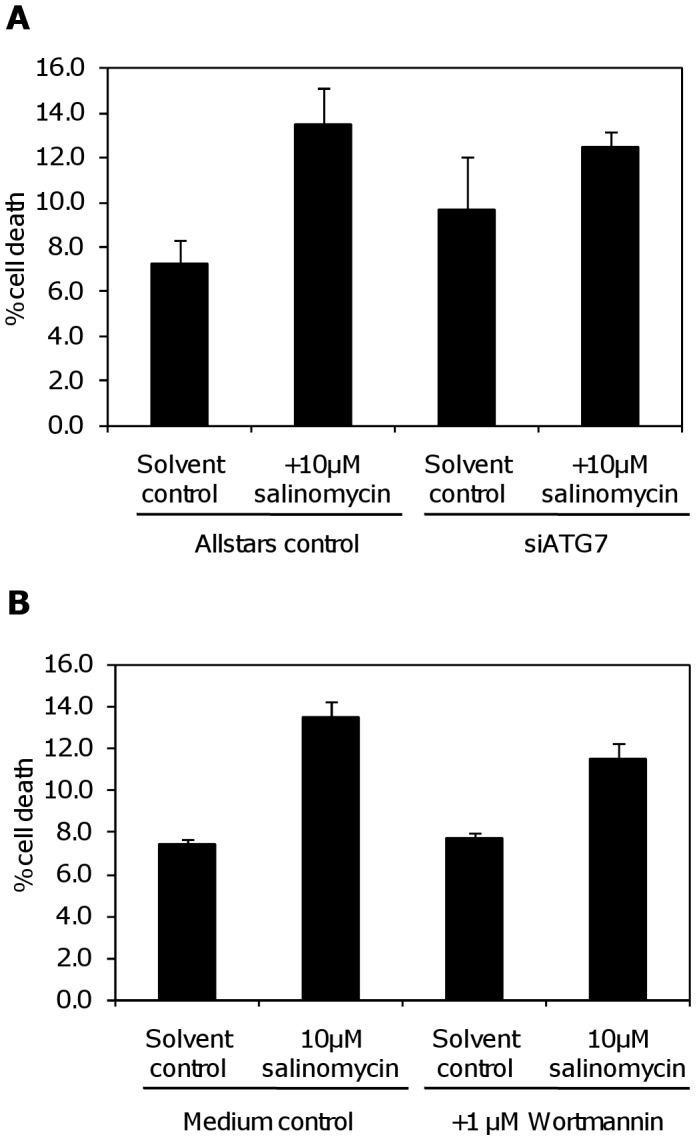
Partial protection against salinomycin toxicity through inhibition of autophagy. Cell death was measured in SW620 by flow cytometry using the ViaCount reagent 24 hours after treatment with 10 µM salinomycin, or solvent control. (**A**) Cells were transfected with siRNA against ATG7, or control oligos, and treated with salinomycin 72 hours after transfection. (**B**) Protection against cell death induction by salinomycin after 3 hours pre-treatment with 1 µM Wortmannin.

At least early after treatment, autophagy is involved in provoking cell death in this system, as inhibiting autophagy through transfection with siRNA against *ATG7* partially prevents cell death 24 hours after salinomycin addition (Figure 5A) in SW620 cells. Pre-treatment with the PI3K inhibitor wortmannin, which is commonly used to inhibit autophagy [16], has an equivalent effect (Figure 5B).

### Salinomycin treatment leads to the induction of reactive oxygen species

**Figure 6:**
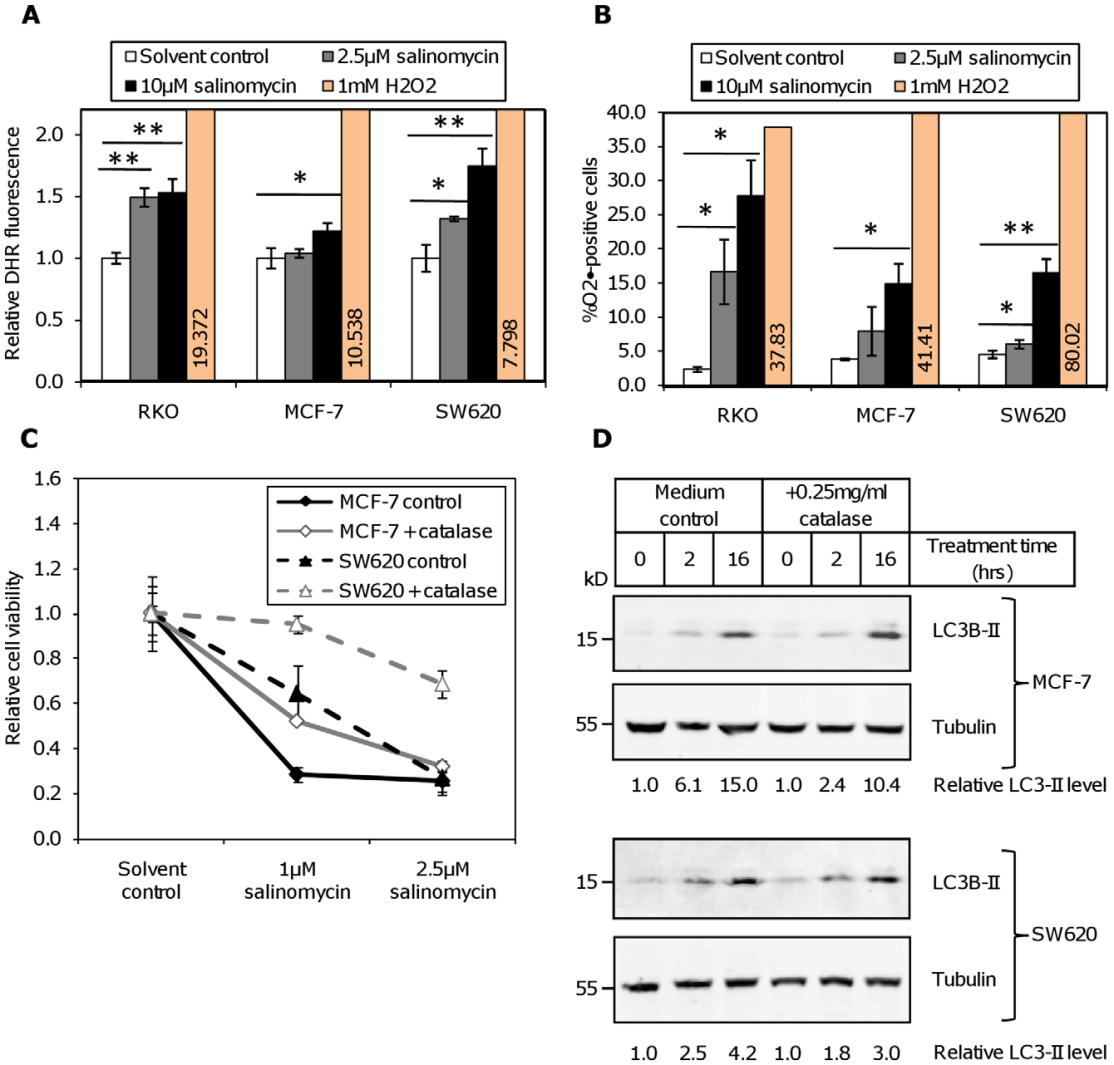
Induction of reactive oxygen species by salinomycin treatment. Production of ROS was measured by flow cytometry in RKO, MCF-7 and SW620 cells 30 hours after treatment with the indicated concentrations of salinomycin or solvent control. Treatment with 1 mM H_2_O_2_ for 1 hour served as a positive control. (**A**) H_2_O_2_ was detected through oxidation of dihydrorhodamine and (**B**) O_2_• through oxidation of dihydroethidium. (*: p<0.05; **: p<0.01, ***: p<0.001 by t-test). (**C**) Effect of catalase on salinomycin toxicity by MTT assay. Cells were pre-treated for 3 hours with 0.25 mg/ml catalase, followed by the addition of the indicated concentration of salinomycin (1 µM; 2.5 µM) or solvent control (ctrl). Absorbance was measured 72 hours after the addition of salinomycin. (**D**) Processing of LC3 in MCF-7 and SW620 after salinomycin treatment in the presence or absence of 0.25 mg/ml catalase. Cells were treated with 2.5 µM salinomycin for the indicated duration; the relative level of LC3-II in comparison to tubulin is indicated below the figure.

Having shown that salinomycin treatment led to the induction of autophagy we opted to elucidate the mechanism which leads to the initiation of salinomycin related autophagy. An increased production of reactive oxygen species (ROS) is often linked to autophagy in combination with loss of the mitochondrial membrane potential (reviewed in ref. [17]). Therefore we measured in the cell lines RKO, SW620, and MCF-7 the production of H_2_O_2_ (Figure 6A) and O_2_• (Figure 6B) after salinomycin treatment. All tested cell lines strongly produced O_2_• in a dose-dependent manner (Figure 6B). RKO and SW620 cells also showed a significant increase in H_2_O_2_ production at both salinomycin concentrations (Figure 6A). In contrast to this finding in MCF-7 there was only a weak formation of H_2_O_2_ and only at the highest salinomycin dose (10 µM).

Pre-treatment with 0.25 mg/ml catalase strongly prevented cell death in the MTT assay in both MCF-7 and SW620 cells (Figure 6C), implicating H_2_O_2_ in cell death in this system. Similar results were obtained with 2 mM N-acetyl cysteine (Figure S3C). Catalase also reduced LC3-processing in MCF-7, SW620 (Figure 6D), and SW480 (Figure 4F), indicating that H_2_O_2_ formation is involved in the autophagic signaling caused by salinomycin treatment.

### Possible involvement of the JNK pathway in induction of autophagy by salinomycin

**Figure 7:**
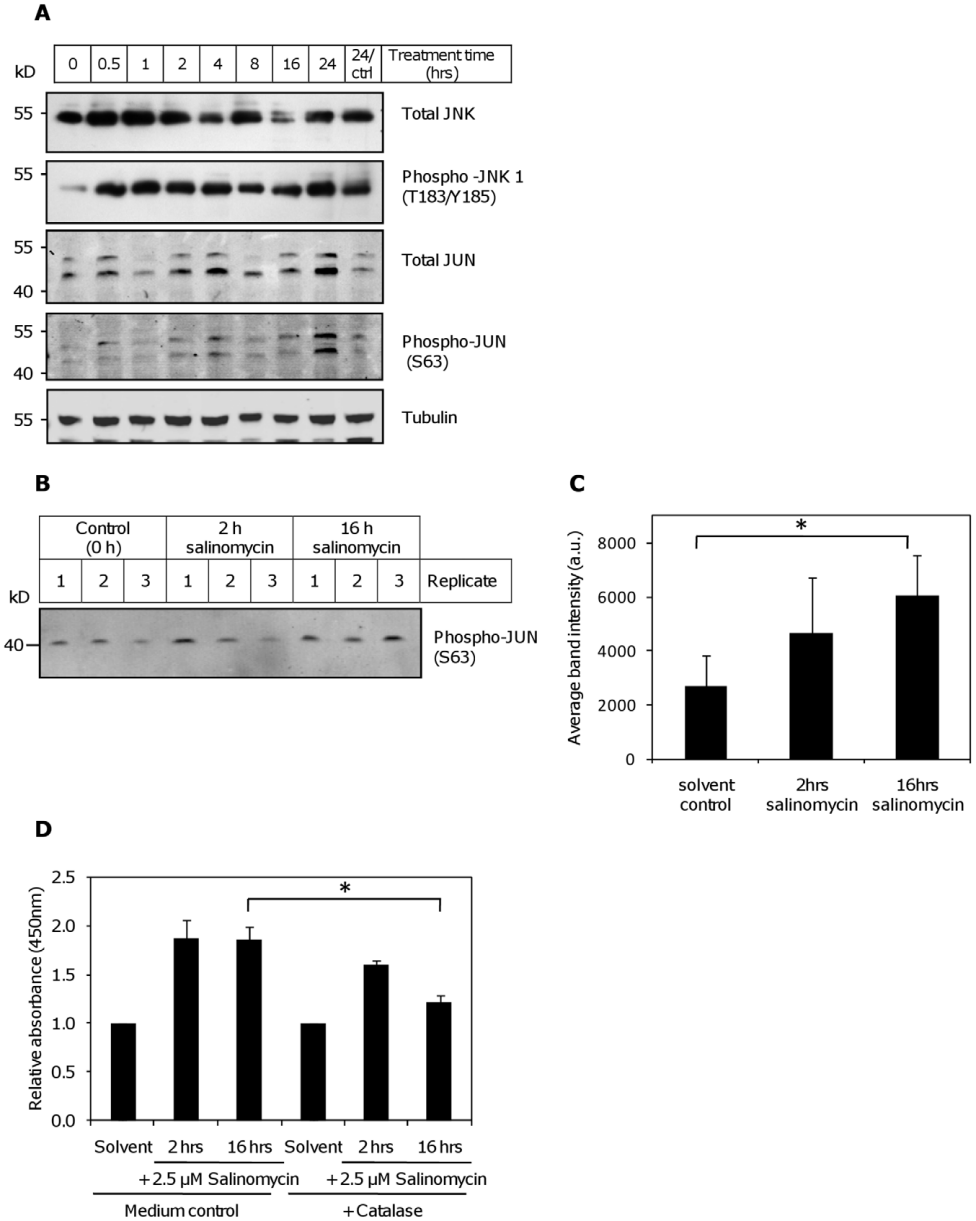
Activation of the JNK pathway by salinomycin. (**A**) Exposure to 2.5 µM salinomycin induced the expression and activation of JNK, as well as its target JUN in MCF-7 cells. (**B**) Increased JNK kinase activity after salinomycin treatment in MCF-7. The JNK kinase assay was carried out after treatment with 2.5 µM salinomycin for the indicated durations; recombinant JUN was used as the substrate. (**C**) Densitometric analysis of the kinase assay. The difference to the untreated control is significant for the 16 hour time point (*: p<0.05). Error bars: standard deviation of three experiments. (**D**) Measurement by ELISA of JNK-phosphorylation after treatment of MCF-7 with 2.5 µM salinomycin, compared to medium control. Cells were pre-treated with 0.25 mg/ml catalase or medium control for 3 hours. Combined results from 2 independent experiments are shown (*: p<0.05).

Reactive oxygen species cause the activation of the MAPK pathways, including JNK [18], [19]. As expected, we observed the induction of JNK phosphorylation in MCF-7 already 30 min after the addition of salinomycin. Its target JUN was induced 24 hours after treatment. At the same time point also the phosphorylation of JUN increased (Figure 7A). In our system, we only clearly detected phosphorylation of the 46 kDa form JNK1 at position T183/Y185, despite stronger expression of JNK2/3. The results of the Western blot experiments were confirmed in a JNK kinase assay in MCF-7, using recombinant JUN as the substrate, which showed a time-dependent increase in kinase activity after salinomycin treatment (Fig. 7B, 7C). The difference to solvent control was significant for the 16 hours time point (p<0.05). Similar results were obtained for SW620 (Figure S3D, S3E).

Confirming that JNK phosphorylation is indeed a consequence of ROS activation, we observed a reduction in phospho-JNK levels in MCF-7 cells after pretreatment with 0.25 mg/ml catalase, which was most pronounced at the 16 hours time point, in comparison to the 2 hours time point. (p<0.05; Figure 7D).

**Figure 8:**
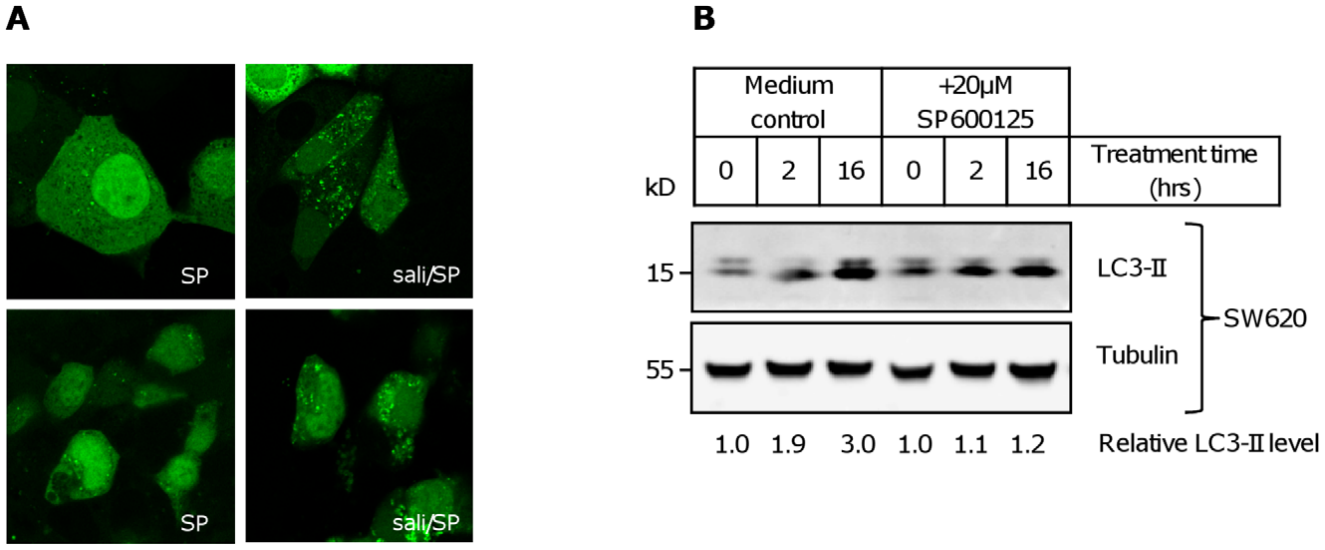
Decreased autophagy induction through salinomycin by inhibition of JNK. (**A**) Confocal microscopy of cells transfected with GFP-LC3 16 hours after treatment as indicated (sali: 2.5 µM salinomycin; SP: 20 µM SP600125). Upper row: MCF-7, bottom row: SW620. (**B)** Effect of pre-treatment with 20 µM SP600125 on LC3 processing after addition of 2.5 µM salinomycin for the indicated durations in SW620 cells. The relative level of LC3-II in comparison to tubulin is indicated below the figure.

JNK signaling appears to be upstream of autophagic signaling in our experiments, as pre-treatment of MCF-7 and SW620 cells transiently transfected with pEGFP-LC3 with 20 µM of the JNK inhibitor SP600125 also led to a decrease of cells with vacuolar staining in combination with a decrease of number of vacuoles per cells and a greater retention of nuclear staining (Figure 8A; comp. Figure 4C). These results were confirmed by Western blot in SW620 (Figure 8B) and SW480 (Figure 4F), where there was a decrease in LC3 processing in comparison to the control protein tubulin when cells were pre-treated with SP600125.

## Discussion

Salinomycin is a 751 Da monocarboxylic polyether antibiotic isolated from a strain of *Streptomyces albus*, which exhibits antimicrobial activity against gram-positive bacteria, mycobacteria, and *Eimeria spp.* [20], [21]. It has also been shown to eliminate leukemia [3] and sarcoma stem cells [22], and other tumor cells exhibiting ABC transporter-mediated multidrug resistance [3]. In spite of its emerging significance as an anti-neoplastic agent, the molecular mechanisms underlying salinomycin induced cell death have received limited attention [2], [3].

In the present study we therefore investigated the mechanisms of caspase dependent and independent cell death mediated by salinomycin in colon and breast cancer cell lines. We report that salinomycin decreased cell viability and colony formation and enriched sub-G1 phase cells in a concentration dependent manner in colon (RKO, SW480, SW620) and breast cancer cell lines (MCF-7, MDA-MB-453, T47D). Interestingly, the investigated CRC cell lines demonstrated greater chemosensitivity to salinomycin than the breast cancer cell lines.

Although all cell lines tested underwent apoptosis as evidenced by a loss of mitochondrial membrane potential, and the appearance of a sub-G1 fraction in the cell cycle profile, only RKO, and to a lesser degree SW480, cells showed a strong activation of both the initiator caspases 8 and 9, and of the effector caspases 3 and 7. Strong caspase 3/7 activation also occurred in the breast cancer cell line MDA-MB-453 at 10 µM salinomycin. A deficient activation of caspase 9 in MCF-7 after DNA damaging treatments despite expression of the pro-caspase and release of cytochrome C into the cytoplasm has been observed earlier [23], [24]. The authors attribute this to the presence of an unknown inhibitor of caspase activity in the cytoplasm, possibly the apoptosis-inhibiting protein XIAP. In SW620, which also do not show significant caspase activation either, down regulation of XIAP could restore caspase activation and sensitivity to TRAIL [25], but whether the same mechanism is also valid for MCF-7 remains to be proven. As the absence of caspase activation in MCF-7 and SW620 did not correspond to a resistance against the toxic effects of salinomycin, cell death must at least in part also proceed via caspase independent pathways in those cell lines. In this respect, it is also notable that caspase activation occurred relatively late even in RKO cells, whereas viability was already clearly decreased 8 hours after treatment. At this time point, only a very weak caspase 3 activation was detectable.

A partial explanation for the differences in sensitivity of the different cell lines may lie in their stem cell properties. Salinomycin has been reported to be more toxic to tumor stem cells [1], [26]. It is interesting to note that SW620 has been found to have more stem cells as defined by CD133/CD44/CD24 expression than SW480 [27]. The high sensitivity of SW620 to salinomycin despite their lack of caspase activity, and low sensitivity to classical inducers of apoptosis like ionizing irradiation and cisplatin [28] is in agreement with these results. Few data are available on the stem cell properties of the RKO cell line, but they have been found to have a similarly high expression level of the stem cell marker NANOG as SW620 [29].

A potential mechanism of caspase-independent cell death is autophagy, which is defined as a controlled lysosomal degradation of macromolecules and organelles. This mechanism is known to initially help cells to survive nutrient deprivation, or hypoxia [30]. Recently, however, several reports have appeared that link autophagy to cell death, e.g. after treatment of mouse fibroblasts and human U937 cells with the caspase inhibitor Z-VAD-fmk [10], [31], or after DNA damage in the absence of BAK and BAX in double knock-out mouse embryonic fibroblasts [32]. Autophagy may generally constitute an alternative cell death pathway in cells with a disrupted apoptotic pathway [33]. Suggestive for autophagic cell death in our work is the appearance of vacuoles in caspase 3-negative MCF-7 cells specifically after salinomycin treatment. Indeed, uptake of monodansyl cadaverine, a marker for autophagy [14], could be shown, especially in MCF-7 and SW620. These results were confirmed by relocalisation of GFP-LC3 and by processing of LC3. Autophagy is known to be induced in MCF-7 cells both after DNA-damage induced by e.g. UV irradiation [23] or application of the topoisomerase I inhibitor camptothecin [24], and after other cell toxic treatments (reviewed in ref. [34]). These cells may constitute a good model system for autophagy due to their defects in caspase activation; there are fewer data available for colon carcinoma cell lines. Sato *et al.* [35] could induce autophagy in SW480, SW620, and several other colon cell lines by amino acid deprivation. SW480 showed a relatively lower level of LC3-II than SW620 though, which is consistent with our data concerning the weaker induction of autophagy as measured by MDC uptake.

The results on MDC uptake and relocalisation of GFP-LC3 were confirmed by Western blot based evidence of LC3 processing to the lipidated form that is typical for autophagy, and increased expression of the autophagy proteins Beclin-1, ATG7, and ATG12. When autophagy was detected by LC3-processing, SW480 showed similar levels of autophagy as SW620. The MDC uptake assay constitutes a convenient screening tool due to its ease of execution, but may not always be sufficiently specific [36]. Other groups have found differences is gene expression between SW480 and SW620 that may both lead to higher as to lower autophagy levels in either cell line. For example, Ghosh et al. (2011) [37] found lower expression of the autophagosomal ubiquitin binding protein p62SQSTM1 in SW620, but SW480 has lower basal expression of BECN1 [38].

Reactive oxygen species have been found to be involved in the autophagic process [5], [39]. In most systems, probably O_2_• is the most important form of free radicals, although experiments in which autophagy was induced by nutrient starvation led to an accumulation of H_2_O_2_ [39]. In our experiments, the main produced ROS was O_2_•; especially MCF-7 cells only showed a minor increase of H_2_O_2_ levels despite clear induction of autophagy. Nevertheless, addition of catalase, a major ROS scavenger, to the cell culture medium partially protected against cell death, and decreased LC3-processing in both SW620 and MCF-7, pointing to the importance of H_2_O_2_ formation in autophagic signaling in our experiments.

ROS can mediate autophagy by activation of the JNK/MAPK pathway; other pathways involve mTOR and HIF1α [32]. One way to activate the MAPK pathways is through the inactivation of MAPK-phosphatases, of which ROS can oxidize the catalytic site cysteine [17]. Another pathway for the activation of JNK proceeds via ASK1 [11]. As JNK activation after salinomycin could in part be inhibited by catalase, it must occur downstream of ROS in our system. In addition, direct inhibition of JNK by SP600125 also reduced formation of autophagic vacuoles and LC3-processing in SW620 and SW480. Activated JNK can promote the transcription of multiple genes involved in the autophagic process, mainly via induction of JUN. Beclin-1 is a direct target of JUN [40], and it has recently been shown by chromatin immunoprecipitation and luciferase reporter assays that also LC3 is a direct JUN target [41]. Inhibition of JNK with SP600125 [6] or by siRNA [42] blocked the induction of ATG7 after treatments which induce autophagy. It is not clear whether this is a direct effect, though. Together, these data point to an important role of the JNK/JUN pathway in the autophagic process. However, as we could only partially block autophagy by JNK inhibition, other signaling pathways must also play a role after salinomycin treatment of cells. The effects of JNK inhibition were also cell line dependent and clearer in the colon cancer cell lines SW620 and SW480. Potential candidates for alternative signaling pathways are ERK1/2 [6], or p38^MAPK^ [43].

In summary, our results demonstrate that salinomycin induces autophagy in breast and colon cancer cell lines, concomitant to the induction of reactive oxygen species. Caspase dependent cell death did also occur in those cell lines in which an intact extrinsic caspase activation pathway exist, but at a later time point and at higher salinomycin concentrations than those which cause significant cell death. Differences in the sensitivity between different cell lines were also not clearly related to differences in caspase activity.

## Materials and Methods

### Cell culture

Colon cancer cell lines RKO, SW480, and SW620, and breast cancer cell lines MCF-7, T47D, and MDA-MB-453 were obtained from ATCC (Manassas, VA, USA). RKO, T47D, and MDA-MB-453 were cultivated in RPMI1640 (Pan Biotech, Aidenach, Germany), supplemented with 10% fetal calf serum (Hyclone-Thermo Scientific, Bonn, Germany) and 100 U/ml penicillin, 100 µg/ml streptomycin (Invitrogen, Darmstadt, Germany); MCF-7, SW480, and SW620 were cultured in DMEM high glucose (Pan Biotech), supplemented with 10% fetal calf serum and 100 U/ml penicillin, 100 µg/ml streptomycin. Salinomycin, doxorubicin, NAC, catalase, wortmannin, and SP600125 were obtained from Sigma-Aldrich (Taufkirchen, Germany).

### MTT assay

Cells were plated at 1500 (SW480 or SW620) or 4000 (all other cell lines) per well in flat-bottom 96-well plates. They were treated with salinomycin at the indicated concentration 16 hours later. Seventy-two hours after this, 5 mg/ml MTT (thiazolyl blue tetrazolium bromide, Carl Roth, Karlsruhe, Germany) in PBS was added per well; cells were lysed after 4 hours by addition of 50 µl triplex solution (10% SDS; 5% isobutanol, 0.012 M HCl). Absorbance was measured at 562 nm. Alternatively, viability was determined with the ViaCount reagent (Millipore, Schwalbach, Germany) on a Guava easyCyte8HT flow cytometer (Millipore), following the instructions of the manufacturer.

### Colony forming assay

Cells were plated at 250 per well in 6-well plates in triplicates in their normal medium; salinomycin and solvent control (DMSO) were added at the time of plating, respectively. Medium was changed to normal growth medium after 5 days. Plates were fixed after 5 more days with 70% ethanol for 20 min at room temperature and stained with 5 mg/ml crystal violet (Carl Roth). Experiments were done in triplicates.

### Flow cytometric measurement of DNA fragmentation

Cells were seeded at 5×10^4^ per well in 6-well plates in triplicate, and treated with salinomycin or solvent control 16 hours later. Forty-eight hours after treatment, cells were harvested by trypsinisation and combined with the floating cells. Cells were fixed in 70% ice-cold ethanol and stained in 0.001% Triton X-100 (Carl Roth), 0.5 mg/ml RNaseA (Sigma-Aldrich), and 60 µg/ml propidium iodide (MP Biochemicals, Illkirch, France) in PBS. Cells were measured on a FACSCalibur flow cytometer (BD Biosciences, Heidelberg, Germany).

### JC-1 staining

For microscopy, cells were seeded on 8-well slides (BD Biosciences), and treated with salinomycin 16 hours later. Cells were stained 48 hours after treatment with 5 µg/ml JC-1 (Axxora, Lörrach, Germany) RPMI 1640 without phenol red (PAA Laboratories, Pasching, Austria)+10% fetal calf serum for 15 min at 37°C, washed twice with PBS, and analyzed immediately. For flow cytometry, JC-1 was added to the cells at a final concentration of 5 µg/ml. Cells were incubated for 10 min at 37°C, after which they were trypsinized and washed extensively with PBS. Treatments were performed in triplicates. Measurements were carried out on a FACSCalibur flow cytometer (BD Biosciences).

### Caspase assays

Caspase activities were measured using the Caspase Glo 3/7, 8, and 9 assays from Promega (Mannheim, Germany), following the instructions of the manufacturer. The resulting luminescence was measured with a Tecan M200 microplate reader (Tecan, Crailsheim, Germany). Values were corrected for differences in cell numbers by staining a parallel plate with 5 mg/ml crystal violet (Carl Roth) after 70% ethanol fixation, extraction of the dye with 1% SDS and measurement of the absorbance at 570 nm.

### Measurement of reactive oxygen species

Generation of H_2_O_2_ was measured by flow cytometry. Cells were incubated at 37°C for 1 hour with 1 µM dihydrorhodamine 123 (DHR; Sigma-Aldrich), washed once with PBS and trypsinized. Cells were counterstained with propidium iodide (60 µg/ml) to exclude non-viable cells. O_2_• radicals were detected by flow cytometry using dihydroethidium (Sigma-Aldrich) following the protocol of Castedo *et al.* [44].

### Staining of autophagic vacuoles by monodansyl cadaverine (MDC) and GFP-LC3 expression

Cells were plated in 12-well plates at a density of 40,000/well, and treated with salinomycin or doxorubicin 16 hours later. Eight hours post treatment, medium was replaced by 1 ml of 100 µM MDC (Sigma-Aldrich) in PBS, and incubated for 15 min at 37°C in the dark. After staining, cells were washed twice with 2 ml of PBS. Subsequently, cells were visualized and photographed in the DAPI channel of the fluorescence microscope (Olympus IX70, Hamburg, Germany); at least 100 cells per well were counted, in triplicates. Alternatively, cells were transfected with pEGFP-LC3 [14] using Fugene 6 (MCF-7) or X-tremeGENE HP (SW620), following the instructions of the manufacturer (Roche, Mannheim, Germany). Twenty-four hours after transfection, the medium was changed and cells were treated for 16 hrs as indicated. Cells were fixed with 4% paraformaldehyde for 10 min at room temperature. Images were recorded by confocal microscopy (Leica TCS SP2, Leica, Wetzlar, Germany) using a 40× objective and 1.0 mm pinhole aperture and a 4× (MCF-7) or 6× (SW620) electronic zoom. Images were processed with the Leica Confocal Software 2.5 build 1227.

### Transfection with siRNA

SW620 cells were seeded at 10^6^ per well in 6 well plates, and transfected with siRNAs against ATG7 (#SI02655373, Qiagen), or Allstars control oligos, labeled with Alexa Fluor 647 (#1027280, Qiagen), using the transfection reagent Attractene (Qiagen) and pUC19 plasmid as carrier DNA, following the instructions of the manufacturer. 48 Hours after transfection, cells were treated with salinomycin or solvent control. The transfection efficiency was determined by flow cytometry.

### Electron microscopy

MCF-7 cells were treated with 10 µM salinomycin for 48 hours. After trypsinization the pellet was fixed in 2.5% glutaraldehyde (Sigma-Aldrich) for 1 hour and post-fixed in 1% osmium tetroxide (Chempur Feinchemikalien, Karlsruhe, Germany) according to Dalton [45] for 20 min at room temperature. After fixation, cells were dehydrated through a graded alcohol series, and embedded in Araldit (Fluka, Taufkirchen, Germany). Ultrathin sections (40 nm) were stained with ultrostain 1 (uranyl acetate) and ultrostain 2 (lead citrate) (Leica). Electron micrographs were recorded using a Zeiss EM 900 (Carl Zeiss, Oberkochem, Germany) electron microscope.

### Western blot, kinase assay and ELISA

2.10^6^ Cells were seeded in 10 cm dishes and treated with salinomycin or solvent control 16 hours later. 0.5–16 Hours after treatment, cells were harvested by scraping into ice-cold RIPA buffer (1% NP40, 0.5% sodium deoxycholate, 0.1% SDS, 150 mM NaCl, 50 mM TrisHCl pH 8.0) containing protease inhibitors (Thermo Scientific) and phosphatase inhibitor cocktail 3 (Sigma,), and sonicated for 15 min with 30 sec pulses (Bioruptor, Diagenode, Liège, Belgium). The protein concentration was measured using the BCA assay (Thermo Scientific) following the instructions of the manufacturer. Incubation with primary antibodies was done overnight at 4°C. Antibodies against Caspase-3 (D175), Pro-caspase 9, JUN, phospho-JUN (S63), JNK, phospho-JNK (T183/Y185), LC3B, Beclin-1, ATG12, and ATG7 (all produced in rabbit) were obtained from Cell Signaling, rabbit-anti-β-actin and mouse-anti-α-tubulin were obtained from Sigma Aldrich, and mouse-anti-cytochrome C was obtained from BD Biosciences.

Signals were recorded with the LI-COR Odyssey system (LI-COR, Bad Homburg, Germany); alternatively, blots were incubated with horse-radish peroxidase labeled secondary antibodies (Promega), the signal was detected using Luminata Forte ECL (Millipore). The SAPK/JNK kinase assay (Cell Signaling) was carried out following the instruction of the manufacturer, using 400 µg of total protein per reaction. Relative intensities of the fluorescence signals obtained by the LI-COR system were determined with ImageJ version 1.43u (NIH, USA). Alternatively, JNK phosphorylation was quantified in 40 µg total protein using the Pathscan phospho-SAPK/JNK (Tyr183/Tyr185) sandwich ELISA kit (Cell Signaling) following the instructions of the manufacturer.

### Statistics

Comparisons between pairs of values were done using the t-test; the Welch t-test was applied if the F-test indicated a significant difference between the variances. Comparisons between multiple groups were done by unifactorial ANOVA, for post-hoc testing between individual pairs of conditions, the Scheffé test was done. Statistical inferences were derived from single experiments.

## Supporting Information

Figure S1 **Effect of salinomycin on cell viability and cytochrome C release.** (**A**) Viability of SW620 after treatment for 24 and 48 hours with the indicated concentrations of salinomycin as determined by the ViaCount assay. (B) Viability of MCF-7 after treatment for 24 and 48 hours with the indicated concentrations of salinomycin as determined by the ViaCount assay. (**C**) Western blot analysis of cytochrome C release. RKO and MCF-7 cells were treated with 5 µM of salinomycin for 48 hours. 0.25 µg/ml Doxorubicin was used as a positive control for cytochrome C release. ERK1/2 was used as internal cytoplasmatic control. (**D**) Relative intensity of cytochrome C signal, in comparison to ERK1/2 as measured with the ImageJ program from the raw intensity data.
(TIFF)

Figure S2 **Effect of salinomycin and doxorubicin on caspase 3/7 activity and viability in RKO cells.** We investigated the impact of these drugs on the induction of apoptosis as measured by capsase 3/7 activity (**A**) and cell viability (**B**) eight, 16, and 24 hours after salinomycin or doxorubicin treatment. The ApoToxGlo Triple assay was used for this, Z-DEVD-aminoluciferin served as the substrate.
(TIFF)

Figure S3 **qRT-PCR analysis of mRNA levels of autophagy-relevant genes, JNK activation, and effect of a free radical scavenger after salinomycin treatment.** (**A**) Induction of *Beclin-1* in MCF-7 cells, 2–16 hours after salinomycin treatment. (**B**) mRNA levels of *ATG7*, *ATG5*, *ATG12*, and *Bcl-2* in MCF-7, 2–16 hours after salinomycin treatment. Open circles: 2.5 µM salinomycin; closed circles: 10 µM salinomycin. *GAPDH* was used as the reference. Measurements were done in duplicates; average values are shown. (**C**) Effect of the free radical scavenger N-acetyl cysteine (NAC) on salinomycin toxicity by MTT assay. Cells were pre-treated for 1 hour with 2 mM NAC, followed by the addition of the indicated concentration of salinomycin or solvent control. Absorbance was measured 72 hours after the addition of salinomycin. (**D**) Increase of JNK phosphorylation (T183/Y185), in comparison to total JNK levels in SW620 after application of 2.5 µM salinomycin for the indicated durations. (**E**) Increased JNK kinase activity after salinomycin treatment in SW620. The JNK kinase assay was carried out after treatment with 2.5 µM salinomycin for the indicated durations; recombinant JUN protein was used as the substrate.
(TIFF)

Materials S1 **Supplementary materials.**
(DOC)
