# Supplementary figures and images for: Salinomycin Induces Autophagy in Colon and Breast Cancer Cells with Concomitant Generation of Reactive Oxygen Species
Source: PLoS One. 2012 Sep 19;7(9):e44132. doi: 10.1371/journal.pone.0044132 (PMC3446972; doi:10.1371/journal.pone.0044132)

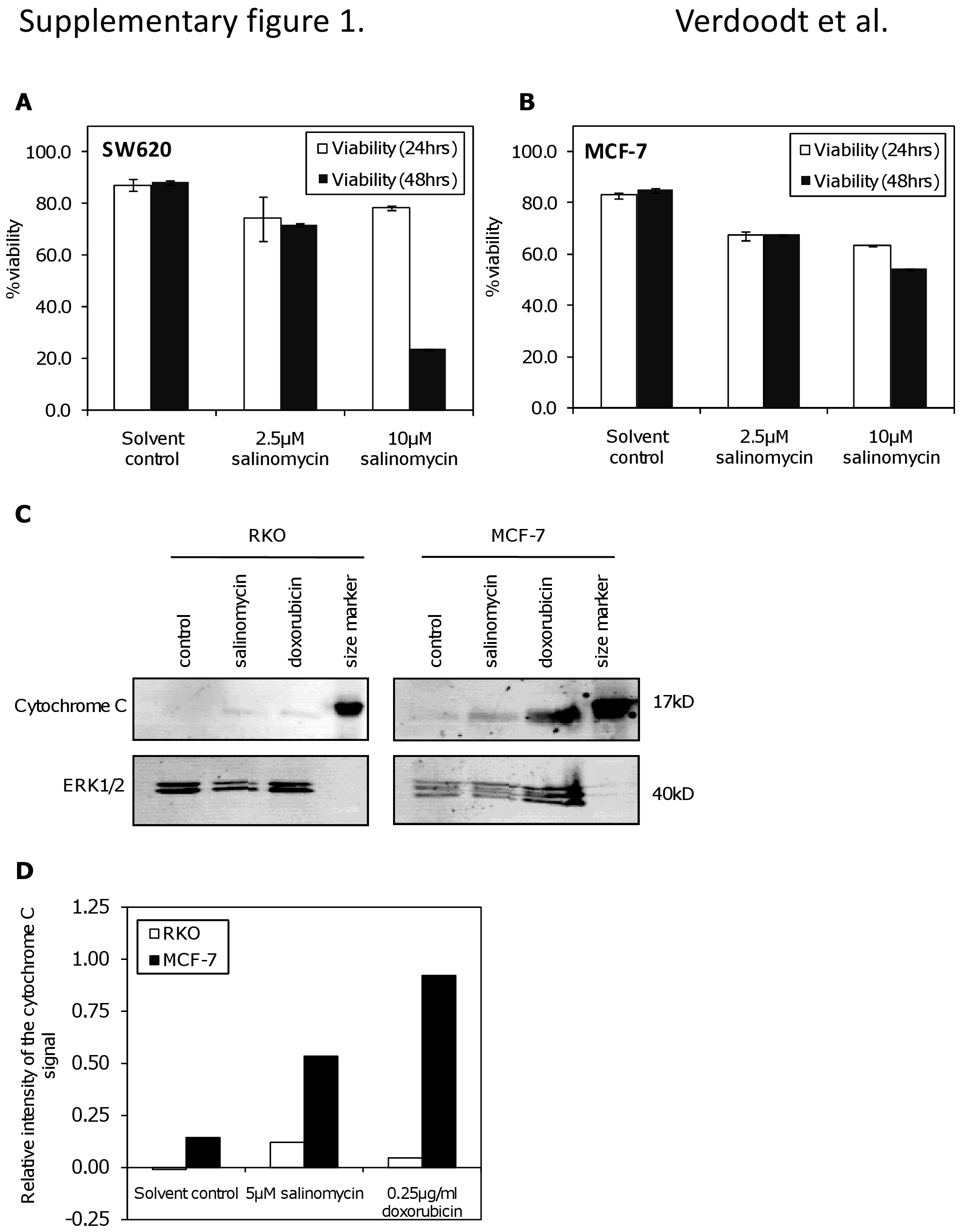

Supplement: Figure S1 — Effect of salinomycin on cell viability and cytochrome C release. (A) Viability of SW620 after treatment for 24 and 48 hours with the indicated concentrations of salinomycin as determined by the ViaCount assay. (B) Viability of MCF-7 after treatment for 24 and 48 hours with the indicated concentrations of salinomycin as determined by the ViaCount assay. (C) Western blot analysis of cytochrome C release. RKO and MCF-7 cells were treated with 5 µM of salinomycin for 48 hours. 0.25 µg/ml Doxorubicin was used as a positive control for cytochrome C release. ERK1/2 was used as internal cytoplasmatic control. (D) Relative intensity of cytochrome C signal, in comparison to ERK1/2 as measured with the ImageJ program from the raw intensity data. (TIFF) [file pone.0044132.s001.tiff]

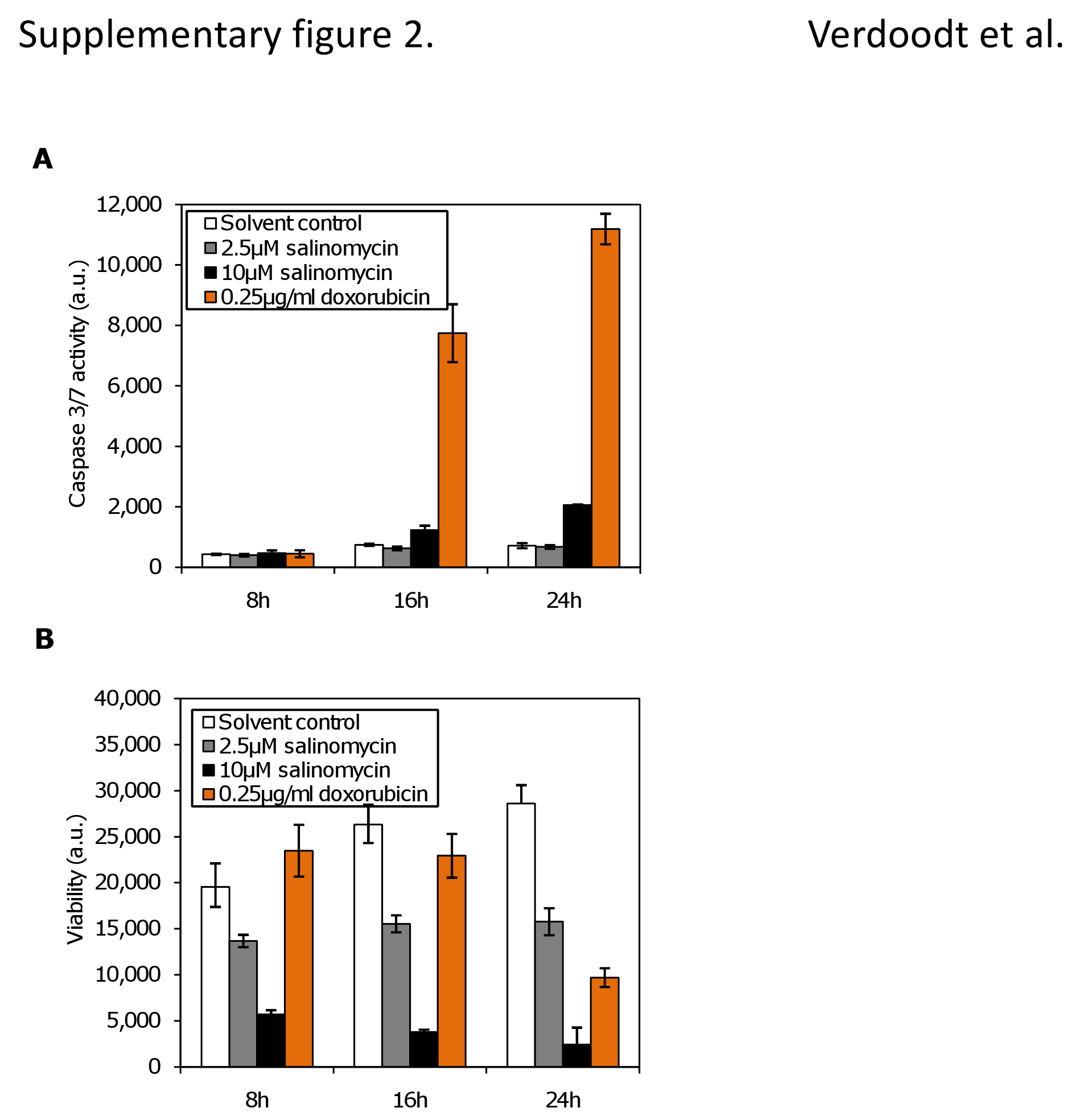

Supplement: Figure S2 — Effect of salinomycin and doxorubicin on caspase 3/7 activity and viability in RKO cells. We investigated the impact of these drugs on the induction of apoptosis as measured by capsase 3/7 activity (A) and cell viability (B) eight, 16, and 24 hours after salinomycin or doxorubicin treatment. The ApoToxGlo Triple assay was used for this, Z-DEVD-aminoluciferin served as the substrate. (TIFF) [file pone.0044132.s002.tiff]

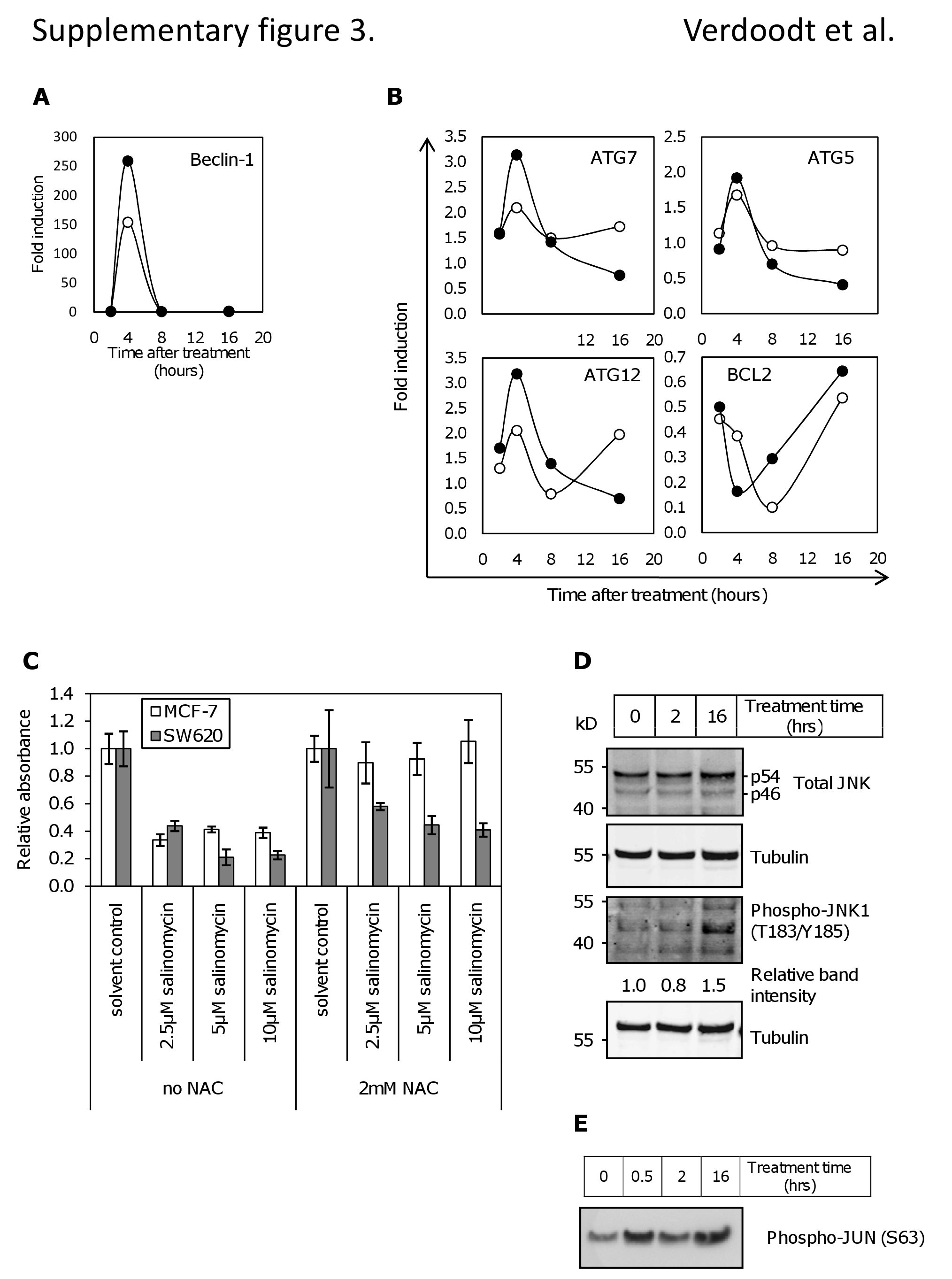

Supplement: Figure S3 — qRT-PCR analysis of mRNA levels of autophagy-relevant genes, JNK activation, and effect of a free radical scavenger after salinomycin treatment. (A) Induction of Beclin-1 in MCF-7 cells, 2–16 hours after salinomycin treatment. (B) mRNA levels of ATG7, ATG5, ATG12, and Bcl-2 in MCF-7, 2–16 hours after salinomycin treatment. Open circles: 2.5 µM salinomycin; closed circles: 10 µM salinomycin. GAPDH was used as the reference. Measurements were done in duplicates; average values are shown. (C) Effect of the free radical scavenger N-acetyl cysteine (NAC) on salinomycin toxicity by MTT assay. Cells were pre-treated for 1 hour with 2 mM NAC, followed by the addition of the indicated concentration of salinomycin or solvent control. Absorbance was measured 72 hours after the addition of salinomycin. (D) Increase of JNK phosphorylation (T183/Y185), in comparison to total JNK levels in SW620 after application of 2.5 µM salinomycin for the indicated durations. (E) Increased JNK kinase activity after salinomycin treatment in SW620. The JNK kinase assay was carried out after treatment with 2.5 µM salinomycin for the indicated durations; recombinant JUN protein was used as the substrate. (TIFF) [file pone.0044132.s003.tiff]
