# Supplementary material for: Salinomycin Induces Autophagy in Colon and Breast Cancer Cells with Concomitant Generation of Reactive Oxygen Species
Source: PLoS One. 2012 Sep 19;7(9):e44132. doi: 10.1371/journal.pone.0044132 (PMC3446972; doi:10.1371/journal.pone.0044132)
Supplement: Materials S1 — Supplementary materials. (DOC) [file pone.0044132.s004.doc]

**Supplementary materials and methods**

Cell fractionation for cytosolic extracts and Western blotting

Trypsinized cells were resuspended in Mito-buffer (20 mM HEPES pH 7.5; 250 mM sucrose; 1mM EGTA; 1 mM EDTA; 10 mM KCl; 1.5 mM MgCl_2_; 1mM DTT; protease inhibitors (Thermo Scientific, Bonn, Germany) and disrupted with 40 strokes in a Dounce homogenizer on ice. The lysate was centrifuged at 800 x g to remove nuclei and remaining intact cells. The supernatant is then spun at 17,000 x g to pellet the mitochondria. The liquid fraction was used for Western blot analysis. Antibodies used were anti-ERK1/2, anti-JNK, anti phospho-JNK, anti-pro-caspase 3 (Cell Signaling, Frankfurt, Germany), anti-Cytochrome C (BD-Biosciences, Heidelberg, Germany), anti-Tubulin (Sigma-Aldrich, Taufkirchen, Germany), anti-Mouse-IRDye 680 (LI-COR, Bad Homburg, Germany), and anti-Rabbit-IRDye 800 (LI-COR). The LI-COR imaging system was used to compare the signal intensity of protein bands. For the caspase immunoblots, cells were harvested by scraping into ice-cold RIPA buffer (1% NP40, 0.5% sodium deoxycholate, 0.1% SDS, 150 mM NaCl, 50 mM TrisHCl pH 8.0) with protease inhibitors (Thermo Scientific), and sonicated (Bioruptor, Diagenode, Liège, Belgium) for 15 min with 30 sec pulses.

**MTT assay**

Cells were plated at 1500 (SW480 or SW620) or 4000 (all other cell lines) per well in flat-bottom 96-well plates. They were treated with salinomycin or solvent control at the indicated concentrations 16 hours later. Where indicated, 2 mM N-acetyl-cysteine (NAC) in PBS was added 1 hour before the salinomycin. Seventy-two hours after this, 5 mg/ml MTT (thiazolyl blue tetrazolium bromide, Carl Roth, Karlsruhe, Germany) in PBS was added per well; cells were lysed after 4 hours by addition of 50 µl triplex solution (10% SDS; 5% isobutanol, 0.012 M HCl). Absorbance was measured at 562 nm. Alternatively, viability was determined with the ViaCount reagent (Millipore, Schwalbach, Germany).

**Caspase 3/7 activity**

The ApoTox-Glo triple assay for caspase 3/7 (Promega, Mannheim, Germany) was used according to the instructions of the manufacturer.

**Real-time qPCR**

Cells were treated for the indicated durations with salinomycin and solvent control, respectively. RNA was isolated using Trizol (Life Technologies, Darmstadt, Germany), following the instructions of the manufacturer. 1000 ng of RNA was used for conversion to cDNA using the Applied Biosystems High Capacity cDNA reverse transcription kit (Life Technologies, Darmstadt, Germany); the equivalent of 20 ng RNA was used per PCR reaction. Quantitative RT-PCR was done on an Eppendorf (Hamburg, Germany) Realplex4 thermal cycler, using Promega GoTaq Master Mix and Qiagen (Hilden, Germany) SYBR green primer assays (ATG12: #QT00035854; ATG5: #QT00073325; ATG7: #QT01879556, Beclin-1: #QT00004221; Bcl-2: #QT00025011). GAPDH (#QT01192646) was used as the reference.
